# Infection and Intoxication through Food

**Published:** 1896-06

**Authors:** 


### INFECTION AND INTOXICATION THROUGH FOOD.

Food may be rendered unfit for consumption from the presence of either pathogenic or toxicogenic bacteria, or the products of their activity, and in the case of animal foods from the existence of disease in the source of supply. Thus, the presence of pathogenic micro-organisms—as of tubercle-bacilli in meat or in milk or butter, of typhoid-bacilli or of cholera-spirilla in milk—renders such food stuffs dangerous by reason of the likelihood of transmitting the specific disease. The presence in food of the micro-organisms of putrefaction on the other hand is capable of occasioning symptoms of varying degrees of intoxication in accordance principally with the virulence of the toxins generated, and in part with the receptivity of the subject. Intoxication or infection may also result from the use as food of the products of diseased animals, *e. g.*, the milk and flesh of cows. It is important to appreciate these several modes of disease-conveyance from both prophylactic and therapeutic considerations. Obviously the flesh or secretions of diseased animals should not be used as food, but the recognition of such conditions is not always possible; so that the thorough cooking of food subserves a salutary purpose over and above that of facilitating digestion. Food-products derived from healthy animals should, of course, be protected from contamination by approved methods.

Much has been added in the last decade to our knowledge of the deleterions effects of contaminated animal products used as food, and a number of poisonous substances have been isolated that seem to be responsible for the symptoms observed. In the class of cases under consideration the post-mortem examination as a rule fails to disclose an adequate cause for death, and the conclusion must be reached that the violent symptoms present, together with the fatal issue, are to be attributed to the action of virulent and fulminant poisons. It is well-known that milk, meat, eggs and fish are all capable of producing such conditions. Recently BROSCH (*Wiener klinische Wochenschrift*, 1896, No. 13, p. 219,) has reported a case in which the evidence seems to show that oysters also are capable of behaving in the same way. An army officer, after partaking of a meal containing a few oysters, was in course of a few hours seized with vomiting, followed by pain in the side and intense headache. As the day progressed, vision became obscure, and there was difficulty in swallowing, with salivation and an inability to pass urine. The right side of the face became paretic and the right pupil widely dilated. Speech was scarcely intelligible, and walking was difficult and unsteady. Cyanosis made its appearance, general muscular relaxation set in and breathing gradually ceased, the heart continuin to beat for two minutes longer. Post-mortem examination revealed hypermia and edema of the brain and spinal cord, with a number of small hemorrhages in the cerebellum and dorsal and lumbar regions. Similar extravasations were present in the pericardium and pleuræ, and beneath the mucus membrane of the epiglottis, of the stomach and of the small intestine. The spleen was enlarged. Immediate histologic investigation disclosed a condition of parenchymatous degeneration of the myocardium, and of the renal epithelium, with a fatty degeneration of the liver-cells. No direct evidence of infection or intoxication could be elicited. It was learned, however, that the man had complained at the time that one of the oysters of which he had partaken was bad. Of a number of persons who had dined with him none presented any untoward symptoms; but he alone had eaten the oysters. The symptoms presented accorded closely with those considered characteristic of fish-poisoning, as observed in unequivocal cases. Of these the most conspicuous was the localized palsy of the muscles of the face; among others was the difficulty in swallowing, the general muscular relaxation, the constipation, the obscuration of vision, and finally the failure of respiration, without actual loss of consciousness, elevation of temperature and pain.

The symptoms detailed characterize especially the cases attended with alkaloidal poisoning; while those dependent upon bacterial invasion are marked rather by gastro-intestinal irritation, diarrhea, and febrile movement. The therapeutic indications would differ widely in the two forms of disease. In the bacterial disorder purgatives and intestinal disinfectants would be required. In the merely toxic condition it were better to empty the stomach and bowels by means of the tube, in conjunction with irrigation. It would further be necessary to evacuate the bladder at frequent intervals, to administer nourishment through the stomach-tube, and to institute artificial respiration, should this function show signs of failure.

—A. A. E. in *Phila. Polyclinic.*

